# Supplementary material for: Patterns of Intron Gain and Loss in Fungi
Source: PLoS Biol. 2004 Nov 30;2(12):e422. doi: 10.1371/journal.pbio.0020422 (PMC532390; doi:10.1371/journal.pbio.0020422)
Supplement: Table S1 — Also available at http://genes.mit.edu/NielsenEtAl/. (4.3 MB ZIP). [file pbio.0020422.st001.zip › NielsenEtAl/html/640.html]

AN7370.1.NCU04396.1.MG09631.1.FG00850.1


```
 CLUSTAL W (1.82) Multiple Sequence Alignments - Introns Inserted


Sequence 1: NCU04396.1	602 aa
Sequence 2: FG00850.1	524 aa
Sequence 3: AN7370.1	592 aa
Sequence 4: MG09631.1	607 aa
Alignment Length: 643 aa
Number Identitical Residues: 89 aa
Alignment Score (without introns) 6352


MG09631.1 	MPSVTMPVRPKQSQSRPPPPAADDTDDDFLVVDDQDGILVIGIDFGTT2FSGVAWATTEE
NCU04396.1	--------------------MPSTPRRSPRKPASSQGTVIIGIDFGTT~FSGVAFTWSNQ
FG00850.1 	--------------------MAAN--------QPLDDTVVVGIDFGTT2FSGVSWAHTRE
AN7370.1  	-----MTFTPSDGILAYRPKIATPSED------DEDDVMVIGIDFGTT2YSGVAWATVAD
          	      .  ....     .  .  .          :. :::******* :***:::   :

MG09631.1 	LEDNPNDINLIMNWPGTGREESKAPSELCYNDDGTIGWGFEVPPEADSIRWFKLLLLRDD
NCU04396.1	VDKIEIITGWDDALSDNTDECKAPTAISYGT-ENKVTWGYSIEPEKEQLKWVKLLLLNDE
FG00850.1 	PDAIEIVTCWDSELNHCSDVEKAPTQLYFDANDHDIKWGYGIPLDKEPLKWFKLLLLDNI
AN7370.1  	FAS--DQINLITSWPGTGREEGKAPTELFYEDD-QIFWGYEIPDDADPVRWFKLLLVKDE
          	                       ..      .:  : **: :  : : ::*.****: : 

MG09631.1 	DMGKELRN~VPQLSDCRDFLKRTGKSAVDVVADYLGMLWKHTIETIVRARGQTVVDAMVI
NCU04396.1	DIPEEVRT~SDKIKEARKYLHKHNKTAEEAVTTFLRHLWEHSIDCITKSISRGMVNYAQF
FG00850.1 	DLPADVAS1ADLLERS------------------------------------------KF
AN7370.1  	DLSEETRS~SEFLLRGRKMLKETGKTAVGLIADYLRLLWAHILESVKKSRGEYVIDALRF
          	*:  :  .    :    .     ..::    :           .    : .    .   :

MG09631.1 	RTVITVPAIWKGYARQAMEKAAKKAGMLEKRPAGPTSLVLANEPEAAALATLIERGRK--
NCU04396.1	HIVITLPAIWPSYAREKMRLAAEQAGMLDRRMGVETQLTFISEPEAAAIATLADMEGRFD
FG00850.1 	QVVLTLPTIWPPYAQNRMKQATQQSGILDGRPAGTTMLQFISEPEAAALATIKDMGKRST
AN7370.1  	HVVITVPAIWKGYARQGMEEAARQAGILSSRPAGETMLSFVPEPEAAALSTLCEPGRR--
          	: *:*:*:**  **:: *. *:.::*:*. * .  * * :  ******::*: :   :  

MG09631.1 	PS~ADSVYIVCDAGGGTV0RLGQDLISYKVEQVNPIS1---------~ALCGGIFIDEAF
NCU04396.1	VK0DEDTFVVVDCGGGTV~D----LISYKLIQAKPMI~VKECVKGSG1GLCGAIFVDDAF
FG00850.1 	VE0TGDTVVVCDAGGGTV0------------------~------GDG1DLCGGVFLDEGF
AN7370.1  	TQ~PGDVYVICDAGGGTV0----DLISYEIASVSPIA~MHEAVEGTG1GLCGGIFIDEAF
          	 .   .. :: *.*****     .  : .  . ..     .. .. .  ***.:*:*:.*

MG09631.1 	ENLCYKQFGLE-WVELSKTAARHIIRDDWEFGIKPQFRKNCASKAFTVRVPSEISFKSAV
NCU04396.1	KEVLKRKFGKR-WEMMDESNRRDMIRTQWETGIKRNFKMG-SDRSYNIRIPMECLLAKER
FG00850.1 	MKLVKEKTPTVSWSSVSKLEEKKFLNDEWEHGIKPQFEN--QMRTWPVYLPGSCNSNSS-
AN7370.1  	EHACKSRLGRR-WDHLSKAGIKEILKGEWEHAIKPQFKPQSSKKEYIVSIPAEAFGKNSL
          	 .    :     *  :.:   :.::. :** .** :*.  .  : : : :* .    .  

MG09631.1 	G-------VKNGRMHFSG~YYCRGAHQG---NVERC2WKGPEQTLLMP-I2AIQT~HVIL
NCU04396.1	R--VIGAITSLPTITITP~GDIKKAFDPVVERINSL~IDGQINAVKEKEG~VNPK0YVIL
FG00850.1 	----ASGLKRRETLDLSS2DEIQSVFLPIAIRIEAL~VRLQVDAIEAKYY~QAPK0YIIL
AN7370.1  	DDMSREPFIKKGRIHFSG2SHIQDAFTESFAGIDKL~VDGQVYKARSQ-G~LDVK0GIIL
          	 .           : ::     : ..      ::                    .  :**

MG09631.1 	VGGLGSSPYLYDYLDEIYAGTGVSILQSTGTKP2RTAICRGAVLKGFLEDMHHPQSS---
NCU04396.1	VGGFGRCNYLYDRVNERFG-AEVEVLQAKGAKP2WTAICRGAVIHAATQSG-VGKLS---
FG00850.1 	VGGFGRSRYLFNHLQGRF---QSTILQSNGNKP2WTAICRGVVVRRLMRYNPSSGLG---
AN7370.1  	VGGLGASPYLYEHLKERHSSAGIIILQSGGMRP~RTAICQGAVYKGFLDGGSDNSGTQQN
          	***:* . **:: :.  ...:   :**: * :*  ****:*.* :            ...

MG09631.1 	-----APVMVTSTVSRTGIGLLMDVPFVHCVHLEEDKKWCPLELNYKASNQVKWFLAK0G
NCU04396.1	-------VQVQTRVARASYGICWNEPWDTQRHNPEDKYWCEIEQKDYARSQMAWLVEE0G
FG00850.1 	-------MVVASRVARMSYGIKYFERFVHGKHKKKDRFWCSKRNSWMANNQMQWFLSE0G
AN7370.1  	GYGVAAPISVTSSISRTSIGIVYHAPFDPAKHLDEDKAWYPDEGEWRANNQLNWYLRR0G
          	. . ::.: * : ::* . *:     :    *  :*: *   . .  * .*: * : . *

MG09631.1 	QDVPECKPVRSPFYHLLYPGQTPRVS--LDLVECERPDAPTRKTS-AV~TPLCTLSFKVD
NCU04396.1	DDITAQEPTSIVIKQNFDRNGPPKTPEDEEIFETNIFPPPTRWDPDVI~KQLCSIKWETV
FG00850.1 	DDISEKRSVHHNYLRLLEGDVFDVS---EEIYCSTSFPPPSRYDGNKV~RSLCNISWNRN
AN7370.1  	DNVSTTDPVRYNFYRLYRKDPGPQFS--VELYECEDHDPPTRLEK-SV0ACLLWNPIYSH
          	:::.   ..     :    .     .   ::       .*:*     :   *        

MG09631.1 	LEREELQEFTNSNCARYHKLSFEVEMIPAGASVEFVVYVDGRKQGARNVNVRFE--
NCU04396.1	IDPSTLPKLKNRVGGVYSELIYRCEMTCSGESMGFAIYRGRKKEGSKSVKVDYNTR
FG00850.1 	VDTKSLPKFTNDYGESFPKLSYRVEMDCEDGIINFAVYSKGRKVAGREVDVQFV--
AN7370.1  	SGGYPSRTLVRATFT-INSPSFEAYAVPAEA-------------------------
          	        : .       .  :.
```
